# A systematic narrative review of the involvement of executive functions in flying performance of pilots

**DOI:** 10.3389/fnrgo.2024.1462304

**Published:** 2024-12-05

**Authors:** Stefanie Enriquez-Geppert, Diede Smit, Marike C. de Boer, Negin Daneshnia, Alex Lafont, Frédéric Dehais

**Affiliations:** ^1^Department of Clinical and Developmental Neuropsychology, University of Groningen, Groningen, Netherlands; ^2^Department of Biomedical Sciences of Cells and Systems, University Medical Center Groningen, Groningen, Netherlands; ^3^Research School of Behavioural and Cognitive Neurosciences, University of Groningen, Groningen, Netherlands; ^4^ISAE-SUPAERO, University of Toulouse, Toulouse, France; ^5^Fédération ENAC ISAE-SUPAERO ONERA, Université de Toulouse, Toulouse, France

**Keywords:** inhibition, working memory updating, shifting, conflicting monitoring, “fly-navigate-communicate”

## Abstract

Piloting is a complex task that demands robust cognitive functions to handle multiple tasks simultaneously in a constantly changing environment. As a result, cognitive abilities, particularly executive functions (EFs), have gained significant importance in relation to flight performance. However, the specific EFs most critical for predicting flight performance remain unclear. Understanding the exact nature of this relationship has the potential to advance research on pilot selection procedures, cockpit design, and influence cognitive training approaches to ultimately improve flight safety. This systematic review aims to pinpoint the most pertinent EFs for various aspects of airplane piloting. A systematic narrative literature review was conducted with a framework focusing on four EFs: working memory updating, set-shifting, response inhibition, and conflict monitoring, as well as three key aspects of flight performance: flying, navigating, and communicating. The findings suggest that multiple EFs predict flight performance outcomes. Notably, working memory updating significantly predicts the management of communication tasks and the making of critical decisions requiring mental flexibility. However, other specific EFs remain understudied. To advance this research area, we recommend conceptualizing EFs and flying measures based on existing theoretical frameworks, using measures sensitive to specific EFs, evaluating flying performance in simulated or real flights, controlling or accounting for factors that affect EFs and flying performance, and investigating the ameliorative potential of EFs with end results on flight performance.

## Highlights

- Conceptualize EFs, use sensitive tasks, and perform cognitive task analysis of flight task.
- Assess flying outcomes in simulated or real flights.
- Control for or take into account factors that have an effect on EFs and/or flying performance.
- Investigate solutions to improve executive functioning.

## Introduction

Flying is a complex task that takes place in a dynamic environment. In familiar settings, much of flying relies on perception-action schemata—cognitive structures that integrate sensory information with motor responses—and automaticity, the ability to perform tasks with minimal conscious effort due to extensive practice and repetition. These processes are acquired through rigorous training and experience. Perception-action schemata and automaticity are fast and occur with little cognitive effort (Schneider and Shiffrin, 1977; Qu et al., 2017). They are primarily stimulus-driven, responding to external stimuli or internal cues rather than by an individual's goals, making them less flexible and more rigid. For example, when taxiing an aircraft on the ground, the pilot automatically steers the aircraft over the runway by coordinating the visual cues of the runway markings with the precise control of the aircraft—a process that requires little cognitive effort. However, in cases of unexpected events or in critical, uncertain environments, such as during landing when abrupt decisions may be required or during adverse weather that demands heightened responsiveness, these schemata and automatic processes may no longer be sufficient. In such situations, more controlled cognitive processes, such as executive functions (EFs) are required to achieve specific goals. EFs play a crucial role in flight phases that require rapid adaptation, such as handling an unexpected go-around decision during descent or processing multiple sources of information during busy air traffic communication exchanges (Dehais et al., 2017). While EFs demand significant cognitive resources, they are essential for safe and adaptive flying, especially when responding to changing environments (for further readings, see Anderson, 2018). In short, both—automatic and controlled—are essential for accurate and safe flying performance, with EFs playing a crucial role in handling complex and dynamic situations (Royall et al., 2002; Causse et al., 2011b).

EFs encompass a set of interrelated top-down processes essential for the regulation and coordination of cognitive activities, e.g., attention and memory, and underly higher-order executive functions such as reasoning (e.g., logical or abstract thinking), problem solving and planning. EFs are particularly critical in situations requiring adaptation to novel, unexpected, or complex challenges where no pre-established schema or automatic responses are available (Lezak, 1995; Shallice, 1988; Diamond, 2013). While there is ongoing debate regarding the exact number and organization of core EFs, this review adapts the “unity and diversity” model, a data-driven model by Miyake et al. (2000), which was further validated by Karr et al. (2018). This model emphasizes both the shared and distinct aspects of EFs. It posits that while there is a common underlying factor (referred to as “common EF”), there are also three separable, core EFs: working memory updating, set-shifting, and response inhibition. In addition, based on follow-up studies, we add a fourth core EF based on neuroscientific results: conflict monitoring (Enriquez-Geppert et al., 2010; Packwood et al., 2011).

Each of this core EFs affects specific flight phases differently: In aviation, working memory updating is considered crucial for assessing flight-relevant aspects, such as evaluating the flight path, fuel availability (Chialastri, 2012), maintaining situational awareness (Sohn and Doane, 2004), as well as managing communication with air traffic control (Morrow and Rodvold, 1993). Set-shifting, or cognitive flexibility, enables pilots to adapt swiftly between tasks, particularly during critical phases like cruising and landing, where unexpected changes—such as shifting weather conditions or congested airspace—demand rapid adjustments in flight path and trajectory (e.g., diversions, go-arounds). Cognitive flexibility is essential in aviation for preventing accidents, as pilots frequently face dynamic environments where cognitive demands can fluctuate sharply. Under high stress, cognitive flexibility may degrade, leading to perseveration, where a pilot continues a suboptimal behavior that may even become maladaptive, as in making unsuitable landing decisions (e.g., Hodgetts et al., 2014; Dehais et al., 2019a). Working memory updating involves refreshing and replacing the information held in working memory. Response inhibition enables the suppression of a dominant or automatic response in favor of a controlled and regulated behavior (Aron, 2007). This skill is essential when routine responses need to be modified, such as when a pilot must halt habitual hand or foot movements during rejected takeoff due to an unexpected event or adapt quickly to a revised missed approach procedure during a go-around. The term inhibition can sometimes be ambiguous, as it is used to describe both reduced performance and the underlying mechanisms behind it. Conflict monitoring, a key part of the broader performance monitoring process, detects conflicts or interference in information processing before a response is executed. It ensures that, when multiple response alternatives are simultaneously are only the appropriate one is selected (Botvinick et al., 2001; Ridderinkhof et al., 2004). For example, conflict monitoring is crucial when pilots must assess and choose between options in cruise phase, such as determining the best course of action during a system failure or a low-fuel situation. In these high-stakes scenarios, pilots need to weigh the risks and benefits of each alternative that are mutually exclusive under significant time pressure. Conflict monitoring is also vital when pilots encounter conflicting information on cockpit displays—such as discrepancies in speed or altitude readings between the pilot's and co-pilot's displays—requiring immediate resolution to maintain safe flight operations. The detection of conflicts triggers compensatory adjustments, for instance by adjusting and increasing attention to task-relevant stimuli (Cak et al., 2019), and/or through the aforementioned inhibition processes of a response. The terms inhibition and conflict monitoring are sometimes used interchangeably or are mixed up. Inhibition is often applied to describe performance outcomes that actually involve different mechanisms, for instance interference resolved with attention recruitment. This is a common challenge in research when it comes to selecting appropriate tasks and interpreting performance results. In sum, these four specific core EFs seem to be important for flying performance, making research into this area relevant. Based on these above examples, the importance of cognitive, and especially executive functioning in the human-technology-environment interaction is becoming increasingly clear.

As aviation technologies become more reliable, we remain confronted with inter-, and intra-individual cognitive differences in pilots, influenced by environmental conditions such as task overload, stress, and fatigue (Dehais et al., 2019a). Currently, professional pilots (civil and military) are selected regarding dexterity, general (e.g., reaction times, short term memory, and attention), specific (spatial attention/mental rotation), and higher cognitive abilities (intelligence, mathematical skills, e.g., Carretta, 2011) using tests like the Computerized Pilot Aptitude Screening System. However, in this selection procedure, EFs are not yet measured directly. In addition, pilots' EFs abilities are not assessed during annual Class 1 medical tests, which could help to detect potential cognitive decline. Understanding the role ofEFs on flying performance can advance knowledge in this support future research. This foundation could guide neuropsychological diagnostics for pilot selection and help the develop methods to enhance EFs in pilots, ultimately improving their flying performance and safety. In this study we systematically review the literature on the relationship between EFs and different aspects of flying performance in pilots using a new framework. Based on the results, we provide concrete suggestions for future research.

## Method

### Literature search and inclusion criteria

For this review, the electronic databases PsycInfo, PubMed, Web of Science, and Cochrane were systematically searched to capture studies on EFs and flying performance. Terms such as “cognition,” “cognitive function^*^,” “cognitive control,” and “executive function^*^” were used to cover core EFs, allowing inclusion of studies on specific tasks and general EF constructs. Aviation-related terms like “flying pilot,” “aircraft pilot,” and “flight simulator” were chosen to ensure relevance to the context. Boolean operators were applied as follows: “OR” was used within each group of related terms to capture a comprehensive range, and “AND” combined the groups, ensuring results included both EF and aviation-related studies. Truncation (using an asterisk*)* was applied to capture word variations. An example search string is*:* “*[(cognition OR cognitive function* OR executive function^*^ OR cognitive control OR human error OR perseveration) AND (flying pilot OR aircraft pilot OR flying performance OR flying error OR flight simulator OR airplane OR commercial flight)].”

**Figure 1:**
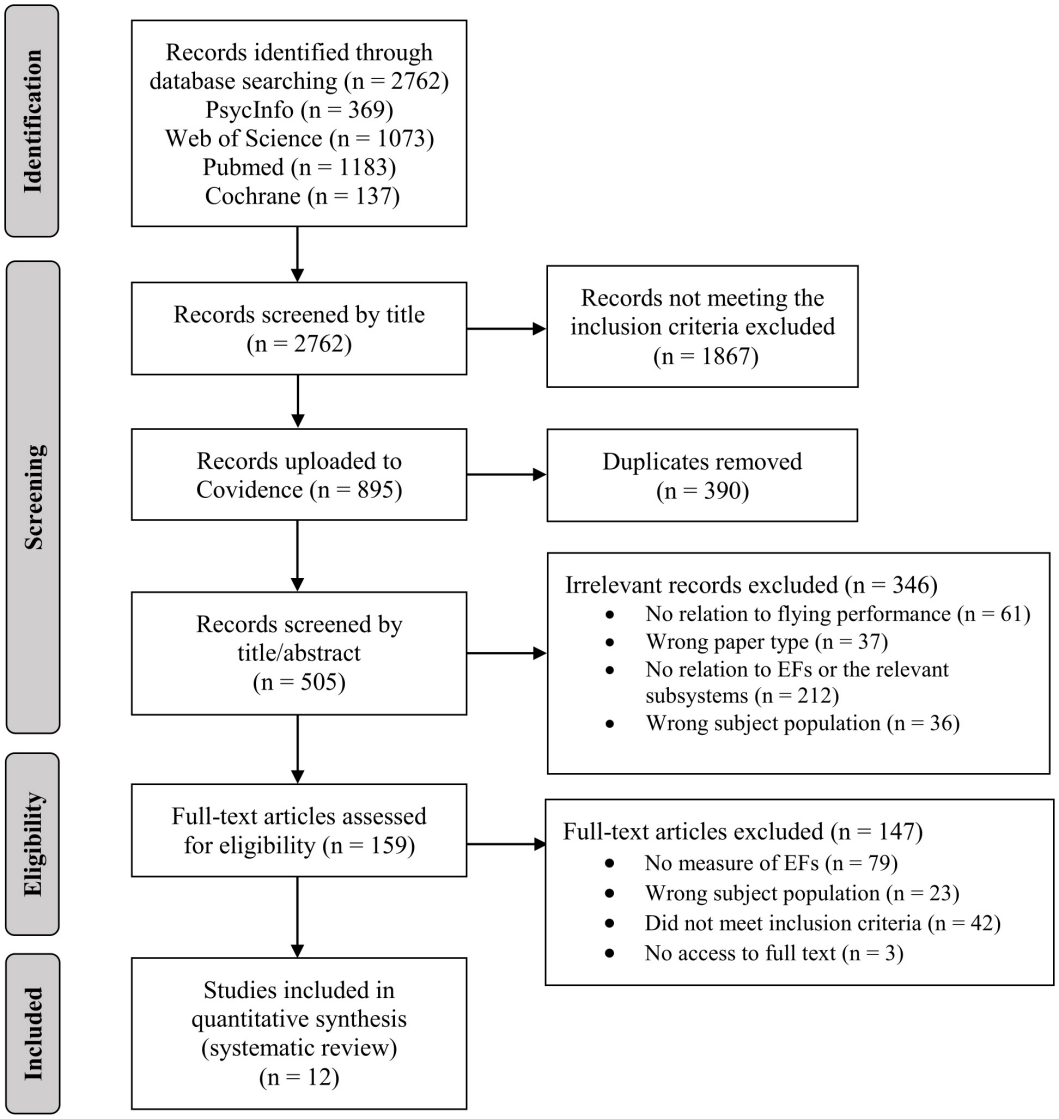
PRISMA flow diagram of the review search.

The search resulted in a total of 1,738 articles (Figure 1), which were screened by title. Articles that did not meet the inclusion criteria were excluded at this point. The remaining articles were uploaded into Covidence (Covidence systematic review software, free version, 2019) and Rayyan (Ouzzani et al., 2016), duplicates were removed, and the abstracts (and if relevant, the full-text) were screened. Exclusion criteria included, for example: no relation to flying performance, the lack of measurements related to EFs (see Figure 1 for the full list in the screening and eligibility phases). The screening process was conducted by a single reviewer trained in EFs research with all exclusions reviewed in consultation with two additional authors to minimize bias. Each study was then carefully read, assessed and verified by three authors to ensure research quality. Eventually, a total of twelve studies were suited for the present systematic review. Data regarding the sample, EFs and flying performance measures, and statistical results were extracted from these studies.

A comprehensive analysis was performed using a framework of EFs and flying performance (EF-Fly) based on two models. First, EFs measures were categorized as either a complex or specific task, following the methodological considerations of Miyake et al. (2000). While specific tasks are designed to measure individual EFs (e.g., the specific n-back tasks for working memory updating), complex tasks (verbal fluency tasks) engage a broader range of cognitive processes, including multiple EFs, general cognition, and non-EFs abilities (Snyder et al., 2015). Since EFs are embedded within specific task contexts, all EF tasks—whether specific or complex—include variability from non-executive processes (non-EFs) associated with a particular task context. For example, in the Stroop task, color processing is such an additional non-EFs that influences performance beyond the core EFs being measured. This means that performance on such tasks, or any associations with flying performance, can also be driven by non-EF processes. When such tasks are used to investigate the relationship with flying performance, the interpretation of statistically significant results is hampered because no conclusion can be drawn as to whether the performance depends on EFs, the cognitive processes controlled by EFs, or both. These complex tasks typically stem from traditional clinical neuropsychology, for example the Wisconsin Card Sorting Test. In the rest of this review, we will use the term “multiple EFs and non-EF abilities” to describe what complex tasks measure. In contrast, the specific tasks often come from cognitive psychology (e.g., Stroop task) and focus on one of the specific core EFs (e.g., conflict monitoring). The advantage here is that conditions are compared that differ primarily in the function of interest. For example, in the Stroop task color words are presented, either written in the same color ink as the presented color word (congruent condition, e.g., the word blue in blue) or in a non-matching ink color (incongruent condition, e.g., the word blue in red). In this latter incongruent condition, participants need to perform a less automated task (naming the color of the word) while there is a more automated task (reading the color word). Thus, this condition triggers an interference between stimulus attributes (color word vs. color of the word) when processing stimulus features (Stroop, 1935). Conflict monitoring then leads to cognitive control and to adjustments in attention away from irrelevant stimuli attributes (color word) to the relevant ones (color of the word) (Botvinick et al., 2001). When comparing the performance between conditions, differences can be attributed to conflict monitoring. This example also illustrates how a specific EF leads to the control of another cognitive process involved in task performance, namely the control of attention triggered by the deception of interference through conflict monitoring. Specific tasks measuring a core EF can be subtests of a larger test battery that may include complex subtests assessing multiple EFs and non-EFs abilities. Table 1 gives an overview of all the EFs measures used in the included studies.

**Table 1:** Overview of EF measures used in the included studies.

Multiple EFs & non-EF		**Computerized Wisconsin Card Sorting Test (WCST)**: In this computerized test participants have to sort cards according to continuously changing criteria (i.e., color, number, or shape). Successful performance on the WCST relies on several complex cognitive processes, such as learning from feedback and strategic planning, but also on specific EFs, such as working memory updating and set-shifting. This test was originally developed to test for frontal lobe dysfunction (e.g., Millner, 1963).
		**Logical Deductive Reasoning Test (LDRT):** This test consists of 24 syllogisms which are based on a logical argument in which one proposition (the conclusion) can be inferred from a rule and from another proposition (Causse et al., 2010). Participants are required to choose one answer from three possible solutions. The test assesses the ability to use structured thinking to deduce a conclusion, involving the isolation and identification of various components of the rule and propositions (i.e., logical reasoning) and verbal working memory.
		**One Touch Stockings task**: This task is a subtest of the Cambridge Neuropsychological Test Automated Battery. In this task participants see two displays containing three colored balls and have to either move the balls in the lower display to copy the pattern shown in the upper display or indicate how many moves are required to copy the pattern. This task is a variant of the Tower of Hanoi and measures multiple EFs and non-EF abilities (e.g., spatial planning) and working memory.
Specific EFs	Working memory (updating)	**Digit Span task:** This task is a subtest of the Wechsler Adult Intelligence Scale - Revised (Wechsler, 1981) and requires participants to verbally repeat a series of digits of increasing lengths, in either a forward (mainly measuring working memory updating) or backward manner (measuring both updating and manipulation of digits in working memory).
		**Two-back task:** In this task participants view a sequence of stimuli and have to determine whether the shape of the current stimulus matches with the stimulus presented two trials earlier. This task is a measure of working memory updating.
		**Computational Span test:** In this test, participants view a sequence of simple arithmetic problems (such as 4–2 = ?, 3 + 3 = ?, 8–6 = ?) and choose the correct answer from three options. They must remember the last digit of each arithmetic problem in the sequence (here, the digits 2, 6, 2). After three correct recalls in a row, the number of arithmetic problems and digits to remember is increased by one (up to a maximum of nine). This test is a measure of working memory updating.
		**Sentence Span Test:** The Sentence Span Test includes a listening and a reading part and measures the ability to simultaneously store and manipulate information in working memory (Stine and Hindman, 1994). This test is a measure of verbal working memory.
		**Spatial Working Memory test:** This test is a subtest of the Cambridge Neuropsychological Test Automated Battery. Participants get to see a varying number of boxes that are randomly distributed on the screen and they have to find a yellow token hidden in one of the boxes by clicking on them. They have to remember which boxes were empty and should not revisit boxes that contained a token in previous trials. The test measures the retention and manipulation of visuospatial information in working memory.
		**Corsi Block-Tapping Test:** Participants observe an experimenter tapping a subset of nine randomly arranged blocks in a specific order. The participant must then replicate the sequence either in forward or reverse order (Arce and McMullen, 2021). This test measures visuospatial working memory updating (Berch et al., 1998).
	Conflict monitoring	**Spatial Stroop task:** The spatial version of this task assesses the conflict or interference between a word identifying a location (“left”) and the actual location where the word is displayed (right side of the screen). Participants have to respond with the appropriate hand according to the meaning of the word, regardless of whether the actual location on the screen is compatible or incompatible with the displayed word.
	Response inhibition	**The Hayling Sentence Completion Test:** Participants need to complete sentences where the last word is missing, but strongly suggested by the context. In the first part participants need to complete the sentence as expected. In the second part participants must generate an unrelated word, requiring inhibition of the automatic response (Belleville et al., 2006; Burgess and Shallice, 1996).
Multiple EFs & non-EF/specific EFs	Test battery	**CogScreen Aeromedical Edition (CogScreen-AE):** This test battery is computer-administered with different subtests, including some neuropsychological tests from the clinical domain (Kay, 1995). Subtests concern different general cognitive functions, including multiple and specific EFs. The battery is comprised of the following subtests: the (a) Backward Digit Span, (b) Math, (c) Visual Sequence Comparison, (d) Symbol Digit Coding, (e) Matching to Sample, (f) Manikin (mental rotation task), (g) Divided Attention Task (simultaneously performing a visual monitoring task and a visual sequence comparison task), (h) Auditory Sequence Comparison, (i) Pathfinder (scanning and connecting a sequence of numbers or letters, and alternating between numbers and letters, similar to the Trail Making Test), (j) Shifting Attention Task (rule-acquisition and application task, similar to the Wisconsin Card Sorting Test), (k) Dual Task (performing a visual motor tracking and a continuous memory task). This test battery was originally developed as part of the medical evaluation for recertification for the evaluation of cognitive changes in pilots with a known or suspected neurological or psychiatric condition.

The table is ordered according to the subdivision into multiple EFs & non EFs and specific EFs. Tasks that are similar, but have different names are marked with the same symbol, for instance both the WCST and the Shifting Attention Task of the CogScreen-AE have the symbol: ⊚ Note that for some tasks, such as the CogScreen subtests, test scores may be calculated based on a number of different items: the number of rule shifts completed, and/or the number of failures to maintain a set, and/or percentage of correct responses.

Second, we classified the different measures according to the golden rule of flying, namely “fly, navigate, and communicate”. This axiom implies a hierarchical categorization in this order (Owens, 2013). The first aspect, flying, is a top priority at all times and relates to flight processes such as the pilot's monitoring and control of pitch altitude and airspeed to achieve and maintain the desired vertical and lateral trajectory. The second aspect is navigating; the pilot needs to know the current location of the aircraft and where it is supposed to be, the weather and potential obstacles. The third aspect is communicating, which follows once flying and navigating is under control and includes communication between pilots, air traffic control, cabin crew or other crew on-board, and the ground crew. The aim of communication is to effectively share goals and intentions, and to enhance situational awareness in order to reduce accident risks (Owens, 2013). Table 2 gives an overview of the flying performance measures used in the included studies.

**Table 2:** Overview of measures of flying performance used in included studies.

Flying	**Flight summary score**: Gives a general overview of important flying aspects. The score is typically provided by a flight simulator and represents the performance on aspects such as scanning the cockpit instruments to detect engine emergencies, accurately executing air traffic control messages, dialling in communication frequencies, staying on course, avoiding other traffic, and executing a visual approach to landing. An overall flight summary score and subscores on the different aspects can be provided. Yesavage et al. (2011) and Kennedy et al. (2013) used the flight summary score based on four components: (a) accuracy of executing air traffic control messages, (b) avoiding other traffic, (c) scanning of the cockpit instruments to detect engine emergencies, and (d) executing a visual approach to landing. Taylor et al. (2000) also used a summary score, based on similar components: (a) staying on course, (b) dialling in communication frequencies, (c) avoiding traffic, (d) monitoring cockpit instruments to detect engine emergencies, and (e) executing a visual landing approach.
	**Flight path deviation (FPD):** Refers to deviations from the ideal flight path during a simulated flight, for instance regarding altitude (vertical axis), speed (horizontal axis), or both.
	**Crosswind landing decision:** Require the pilot to decide whether the meteorological conditions and the aircraft's maximum crosswind limit at the time of the approach are compatible with landing or requires a go-around and diversion. The outcome is usually a binary variable: the pilot either correctly decides to go-around or erroneously tries a landing (correct/incorrect).
	**Flight ability** is assessed during a simulated 5-hour airplane panel task, with flight instructors evaluating participant performance, although the assessment criteria are not clearly defined.
Navigating	**Diversion management/response:** Pilots receive an unexpected instruction from air traffic control telling them to divert from their original plan. They have to independently locate and safely fly to another airport and orbit there at a specific altitude. The diversion management score is calculated based on the speed of determining a new flight plan and maintaining accurate situational awareness while continuing to fly and communicate with air traffic control.
	**Staying on course:** A subscore of the flight summary score and is an automatic index generated by a flight simulator.
	**Alternate aerodrome errors:** Based on not flying to the alternate aerodrome and not following the orbiting procedures.
Communicating	**Aviation communication*****:*** Typically assessed with tasks utilizing air traffic control messages. For instance pilots are asked to read back (repeat) the instructions of each message and execute them. Instructions can pertain to a variety of tasks, including initiating a new flying course, changing the radio frequency, or changing the transponder code. These tasks can also be applied outside a flight simulator or aircraft. In that case pilots usually have to listen to air traffic control messages describing an aircraft's route while looking at a chart of an airspace. The pilots have to read back the message, answer questions about the current flight position and route of the aircraft, and/or recall the route by drawing it on the chart.

**Figure 2:**
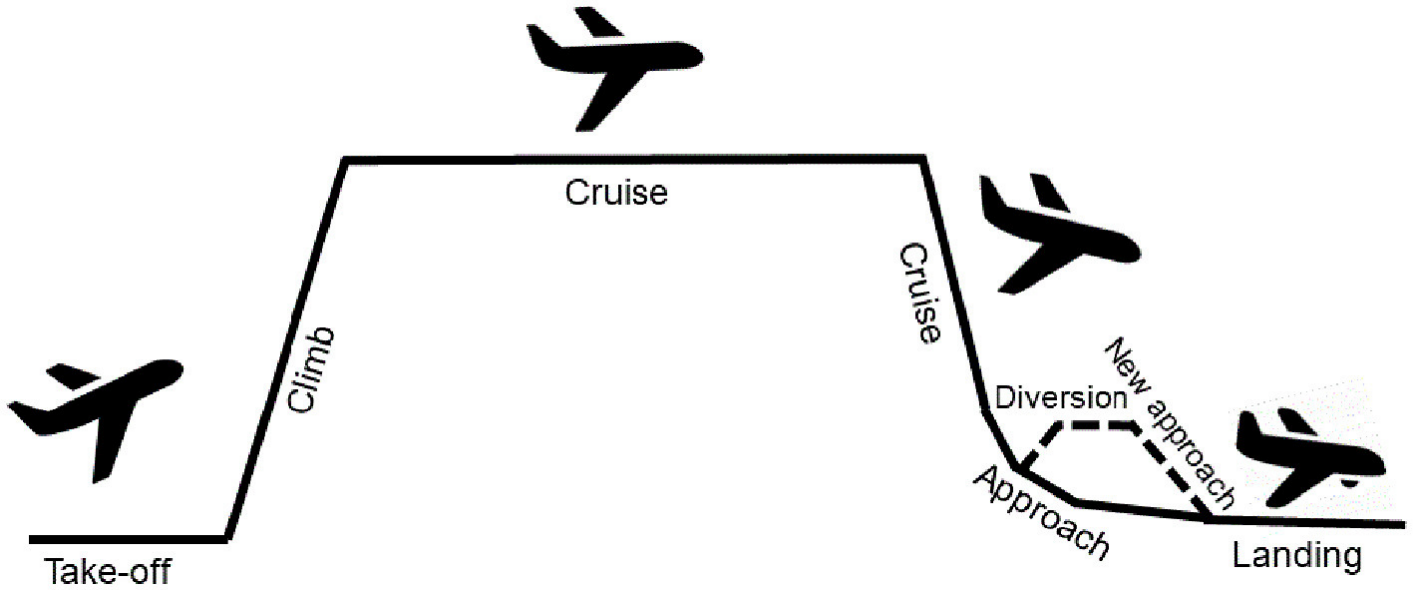
Flight phases adapted after Keller et al. (2003).

Although flying performance measures can be categorized as flying, navigating, or communicating, there are major differences even within the same category. For example, some of the outcome measures relate to specific flight phases (e.g., visual landing approach), while others are assessed in different flight phases (e.g., communication). In addition, workload may differ between the flight phases (e.g., communication during cruising vs. approach) (see Figure 2 for an overview of the flight phases). The used measures may also represent outcomes of different flight tasks (e.g., flight path deviation as a result of monitoring and adapting the flight path) that often need to be performed while performing other flight tasks (e.g., monitoring and adapting the flight path deviation while communicating with air traffic control during approach) and therefore also differ in workload. This can lead to differences in performance, e.g., in avoiding traffic and flight path deviations during final landing approach compared to the cruise phase (Flight Safety Foundation, 2014).

## Results and discussion

### Synthesized findings

Data was extracted from the twelve eligible studies and qualitatively evaluated. In total 856 pilots of different ages were included across the twelve studies, with flying experience extending from 0 to 19033 h. Expertise levels ranged from student pilots, to private licensed pilots under visual flight rules, to instrument rated and commercial pilots, as well as pilot instructors. In all studies, except the ones from Morrow et al. (2003) and Zheng et al. (2022), flying performance was assessed in a flight simulator (e.g., Frasca 141 or concerted Cessna 172) with assorted features (with or without motion vibration, sound elements, fixed-wing, etc.). The included studies mainly used complex tasks (involving EFs and other cognitive abilities), and some also included tasks measuring specific EFs. The majority of the studies reported correlation coefficients between different EFs measures and measures of flying performance. Other approaches included for instance regression designs and took multiple (cognitive) predictors into account. In this review, we report all statistically significant and non-significant values obtained from the included studies. If the values are not listed here, they were not reported in the original study. The sample sizes of the studies are only reported the first time results from that specific study are mentioned. Due to a lack of the necessary parameters and substantial heterogeneity across the included studies, a meta-analysis was not possible.

In the coming sections, we report for each specific aspect of flying performance the results from the included studies, discuss the main findings, and finally provide suggestions for future research. Table 3 provides an overview of the results.

**Table 3:** Overview of the cognitive functions and flight performances measured in each study, along with the key statistical results.

**References**	**Task**	**Cognitive function**	**Flight performance**	**Analysis**	**Results**
Zheng et al. (2022)	Hayling Sentence Completion Task	Inhibition	Flying: flight ability	Correlations	(*r* = −0.40, *p* < 0.05)
	Corsi Block Test	Working memory updating	Flying: flight ability	Correlations	(*r* = −0.29, *p* > 0.05)
	WCST	Multiple EFs and non-EFs	Flying: flight ability	Correlations	(*r* = −0.14, *p* > 0.05)
Van Benthem and Herdman (2021)	CogScreen-AE: Shifting Attention Task composite score	Multiple EFs and non-EFs	Navigating: diversion response	Correlations	(*r* = −0.213, *p* > 0.05)
	CogScreen-AE: Shifting Attention Task composite score	Multiple EFs and non-EFs	Navigating: alternate aerodrome errors	Correlations	(*r* = −0.271, *p* < 0.05)
Taylor et al. (2000)	CogScreen-AE: General Speed/WM composite score	Multiple EFs and non-EFs	Flying: flight summary score	Correlations	(*r* = 0.57, *p* < 0.0001)
	CogScreen-AE: General Speed/WM composite score	Multiple EFs and non-EFs	Navigating: staying on course	Correlations	(*r* = 0.49, *p* < 0.05)
	CogScreen-AE: General Speed/WM composite score	Multiple EFs and non-EFs	Aviation communication: dialing in frequencies	Correlations	(*r* = 0.39, *p* < 0.05)
	CogScreen-AE: Shifting Attention Task composite score	Multiple EFs and non-EFs	Flying: flight summary score	Correlations	(*r* = 0.41, *p* < 0.05)
	CogScreen-AE: Shifting Attention Task composite score	Multiple EFs and non-EFs	Navigating: staying on course	Correlations	(*r* = 0.23, *p* < 0.05)
	CogScreen-AE: Shifting Attention Task composite score	Multiple EFs and non-EFs	Aviation communication: dialing in frequencies	Correlations	(*r* = 0.39, *p* < 0.05)
Kennedy et al. (2013)	Cogscreen-AE: Pathfinder composite score	Multiple EFs and non-EFs	Flying: flight summary score	Linear mixed effects model	(β = −0.194, *p* < 0.0001)
	Cogscreen-AE: Pathfinder composite score	Multiple EFs and non-EFs	Aviation communication	Linear mixed effects model	(β = −0.266, *p* < 0.0001)
	CogScreen-AE: Shifting Attention Task composite score	Multiple EFs and non-EFs	Flying: flight summary score	Linear mixed effects model	(β = 0.062, *p* = 0.0212)
	CogScreen-AE: Shifting Attention Task composite score	Multiple EFs and non-EFs	Aviation communication	Linear mixed effects model	(β = 0.095, *p* = 0.0208)
Yesavage et al. (2011)	CogScreen-AE: Executive function composite score	Multiple EFs and non-EFs	Flying: flight summary score	Linear mixed effects model	(β = 0.065, *p* = 0.188)
Causse et al. (2011a)	WCST	Multiple EFs and non-EFs	Flying: Flight Path Deviation (FPD)	Correlations	(*r* = 0.25, *p* > 0.05)
	WCST	Multiple EFs and non-EFs	Flying: Crosswind Landing Decision (CLD)	Discriminant analysis	[β = −0.379, *F*_(1, 14)_ = 2.584, *p* = 0.142]
	LDRT	Multiple EFs and non-EFs	Flying: Flight Path Deviation (FPD)	All possible subset regression analysis	[*F*_(1, 15)_ = 9.20, *p* = 0.0083]
	LDRT	Multiple EFs and non-EFs	Flying: Flight Path Deviation (FPD)	Correlations	(*r* = −0.63, *p* < 0.01)
	LDRT	Multiple EFs and non-EFs	Flying: Crosswind Landing Decision (CLD)	Discriminant analysis	[β = −0.144, *F*_(1, 14)_ = 0.486, *p* = 0.503]
	2-back Task	Working memory updating	Flying: Flight Path Deviation (FPD)	All possible subset regression analysis	[*F*_(1, 15)_ = 5.08, *p* = 0.0395]
	2-back Task	Working memory updating	Flying: Flight Path Deviation (FPD)	Correlations	(*r* = −0.35, *p* > 0.05)
	2-back Task	Working memory updating	Flying: Crosswind Landing Decision (CLD)	Discriminant analysis	[β = 1.551, *F*_(1, 14)_ = 20.676, *p* = 0.001]
	Spatial Stroop Task	Conflict monitoring	Flying: Flight Path Deviation (FPD)	Correlations	(*r* = 0.15, *p* > 0.05)
	Spatial Stroop Task	Conflict monitoring	Flying: Crosswind Landing Decision (CLD)	Discriminant analysis	[β = 0.264, *F*_(1, 14)_ = 1.072, *p* = 0.327]
Causse et al. (2011b)	WCST	Multiple EFs and non-EFs	Flying: Flight Path Deviation (FPD)	Experience-partialled correlations	(*r* = −0.23, *p* = 0.452, *R*^2^ = 0.05)
	WCST	Multiple EFs and non-EFs	Flying: Crosswind Landing Decision (CLD)	One-way ANOVA	[*F*_(1, 30)_ = 5.33, *p* = 0.027, ηp^2^ = 0.15]
	LDRT	Multiple EFs and non-EFs	Flying: Flight Path Deviation (FPD)	Experience-partialled correlations	(*r* = −0.54, *p* = 0.006, *R*^2^ = 0.30)
	2-back Task	Working memory updating	Flying: Flight Path Deviation (FPD)	Experience-partialled correlations	(*r* = −0.41, *p* = 0.022, *R*^2^ = 0.17)
	2-back Task	Working memory updating	Flying: Crosswind Landing Decision (CLD)	One-way ANOVA	[*F*_(1, 30)_ = 9.76, *p* = 0.003, ηp^2^ = 0.25]
	Spatial Stroop Task	Conflict monitoring	Flying: Flight Path Deviation (FPD)	Experience-partialled correlations	(*r* = 0.19, *p* = 0.322, *R*^2^ = 0.03)
Van Benthem and Herdman (2016)	CogScreen-AE: Speed/WM composite score	Multiple EFs and non-EFs	Flying: Flight Path Deviation (FPD)	Correlations	(*r* = −0.456, *p* < 0.01)
	CogScreen-AE: Speed/WM composite score	Multiple EFs and non-EFs	Flying: Flight Path Deviation (FPD)	Multiple regression analysis	(β = −0.337, *t* = −3.000, *p* = 0.004)
	CogScreen-AE: Shifting Attention Task composite score	Multiple EFs and non-EFs	Flying: Flight Path Deviation (FPD)	Correlations	(*r* = −0.324, *p* < 0.05)
	CogScreen-AE: Shifting Attention Task composite score	Multiple EFs and non-EFs	Flying: Flight Path Deviation (FPD)	Multiple regression analysis	(β = −0.216, *t* = −2.207, *p* = 0.032)
Causse et al. (2017)	One Touch Stockings of Cambridge	Multiple EFs and non-EFs	Flying: Flight Path Deviation (FPD)	Correlations	(*r* < 0.40)
	Spatial Working Memory Test	Working memory updating	Flying: Flight Path Deviation (FPD)	Correlations	(*r* < 0.40)
Van Benthem and Herdman (2017)	CogScreen-AE: Shifting Attention Task composite score	Multiple EFs and non-EFs	Navigating: diversion management	Correlations	(*r* = −0.527, *p* < 0.01)
Taylor et al. (2005)	Sentence Span Test, Computation Span Test, Digit Span Task	Working memory updating	Aviation communication	Correlations	(*r* = 0.76, *p* < 0.05)
	CogScreen-AE: Shifting Attention Task composite score	Multiple EFs and non-EFs	Aviation communication	Correlations	(*r* = 0.43, *p* < 0.05)
Morrow et al. (2003)	Sentence Span Test	Working memory updating	Aviation communication: ATC message readback	Hierarchical Regression Analysis	(β = 0.31, *p* < 0.001)
	Sentence Span Test	Working memory updating	Aviation communication: ATC message readback	Correlations	(*r* = 0.42, *p* < 0.001)
	Sentence Span Test	Working memory updating	Aviation communication: Route probe	Hierarchical Regression Analysis	(β = 0.34, *p* < 0.001)
	Sentence Span Test	Working memory updating	Aviation communication: Route probe	Correlations	(*r* = 0.40, *p* < 0.001)
	Sentence Span Test	Working memory updating	Aviation communication: route recall	Hierarchical REGRESSION ANALYSIS	(β = 0.38, *p* < 0.001)
	Sentence Span Test	Working memory updating	Aviation communication: route recall	Correlations	(*r* = 0.46, *p* < 0.001)

#### Flying

##### Results: Flight summary score and multiple EFs and non-EF abilities

Taylor et al. (2000) evaluated the associations between five cognitive composite scores and an overall flight summary score in 100 civilian pilots. The flight summary score was based on five components: (a) staying on course, (b) dialling in communication frequencies, (c) avoiding traffic, (d) monitoring cockpit instruments to detect engine emergencies, and (e) executing a visual landing approach. The composite score, based on a combination of CogScreen-AE subtests and the Shifting Attention Task composite score, measures multiple EFs and non-EF abilities. The CogScreen-AE subtests composite score, together with the three other non-EFs cognitive composite scores, was a significant predictor in a multiple regression model explaining 45% of the variance [*B* = 0.31, *p* = 0.0001, *F*_(1, 81)_ = 20.62]. The Shifting Attention Task composite score was not a significant predictor, most likely due to its correlation with the other non-EFs cognitive factors. Additionally, both the CogScreen-AE subtests composite score (*r* = 0.57, *p* < 0.0001, *R*^2^ = 0.33) and the Shifting Attention Task composite score (*r* = 0.41, *p* < 0.05) showed a significant correlation with the overall flight summary score.

Kennedy et al. (2013) investigated whether four cognitive composite scores, together with the level of expertise, could predict an overall flight summary score in 236 certificated pilots. The flight summary score was based on: (a) accuracy of executing air traffic control messages, (b) avoiding other traffic, (c) scanning of the cockpit instruments to detect engine emergencies, and (d) executing a visual approach to landing. The cognitive composite score based on a combination of CogScreen-AE subtests, the Shifting Attention Task composite score, and the Pathfinder composite score were all measures of multiple EFs and non-EF abilities. The fourth composite score was a measure of processing speed. A mixed effects model with the predictors: Shifting Attention Task composite score, Pathfinder composite score, processing speed, and expertise was set up. The composite score based on CogScreen-AE subtests was not included in the model, as it significantly correlated with the Pathfinder composite score and was not correlated with the flight summary score. The results showed that both the Shifting Attention Task and Pathfinder composite scores were significant predictors of the flight summary score (β = 0.062, *p* = 0.021 and β = −0.194, *p* < 0.0001, respectively), together with processing speed and expertise.

The study by Yesavage et al. (2011) assessed seven different cognitive composite scores in a subsample of 86 private licensed pilots. They examined if these composite scores based on unspecified combinations of CogScreen-AE subtests could predict performance decline in a flight summary score over the course of 10 years. The flight summary score was based on the same four components as the study by Kennedy et al. (2013) described above. The cognitive composite scores were: *Executive function, Symbol-digit recall, Working memory updating, Working memory manipulation, Processing speed, Motor coordination*, and *Tracking*. The composite score *Executive function* is considered to be a measure of multiple EFs and non-EF abilities. Higher scores on *Executive function*, together with a faster *Processing speed*, predicted a slower rate of decline in the flight summary score in a receiver operating characteristic (ROC) analysis (K = 0.19, χ2 = 4.55, *p* < 0.05), which was confirmed with a random-effect linear growth model. *Executive function* did not significantly predict initial performance on the flight summary score (β = 0.065, *p* = 0.188). The composite scores *Working memory updating and Working memory manipulation* are also considered as measures of multiple EFs and non EFs since the CogScreen-AE subtests are unspecified. These composite scores did not predict the rate of decline of an overall flight summary score in an ROC analysis over the course of 10 years.

###### Discussion flight summary score

The studies by Taylor et al. (2000), Kennedy et al. (2013), and Yesavage et al. (2011) show mixed results on the involvement of multiple EFs and non-EF abilities in the performance on an overall flight summary score. Since the used complex tasks and flight summary scores are very broad measures of general cognitive abilities and different flying skills, respectively, and operationalised in very different ways, the results of these studies are not very conclusive.

###### Comments for future studies

An important next step is to assess the contribution of specific EFs (i.e., specific tasks measuring working memory updating, set-shifting, response inhibition, conflict monitoring) to flying as measured by a flight summary score. To facilitate the interpretation of the significance of the predictors, it is recommended to report partial or semipartial correlations. Additionally, the results emphasize the importance of controlling for or taking into account factors affecting flying performance. For instance in a multiple regression model, add flight experience or expertise as a predictor, in future studies to examine EFs independent of experience. Alternatively, stratified sampling can be used to ensure level of experiences are evenly distributed, minimizing possible confounding effects.

##### Results: Flight path deviation and multiple EFs and non-EF abilities

Four studies looked into the relationship between FPD and multiple EFs and non-EF abilities. Causse et al. (2011a) assessed the EFs abilities of 24 private licensed pilots in relation to their FPD during take-off and reaching a specific waypoint. Multiple EFs and non-EF abilities, as measured by the number of perseverative errors on the computerized WCST, did not predict FPD in a multiple regression analysis, and showed a non-significant correlation with FPD (*r* = 0.25). Causse et al. (2011b) did a highly similar study in 32 private licensed pilots. Here, they additionally controlled for level of experience. Again, a non-significant correlation between FPD and multiple EFs and non-EF abilities, as measured by the number of errors on the computerized WCST, was found (*r* = −0.23, *p* = 0.452, *R*^2^ = 0.05). In contrast, the two above-mentioned studies did find significant effects of logical reasoning performance on FPD, which is also considered to be part of multiple EFs and non-EF abilities. Causse et al. (2011a) found that logical reasoning performance on the LDRT was a significant predictor in a multiple regression analysis [*F*_(1, 15)_ = 9.20, *p* = 0.008]. In this analysis, working memory updating and experience were also significant predictors. Additionally, they found a significant negative correlation between logical reasoning ability and FPD *(r* = −0.63, *p* < 0.01). Causse et al. (2011b) confirmed these results and reported a negative moderate experience-partialled correlation between logical reasoning performance on the LDRT and FPD (*r* = −0.54, *p* = 0.006, *R*^2^ = 0.30). Both studies found that better logical reasoning abilities were associated with a smaller FPD during take-off and reaching a specific waypoint.

Van Benthem and Herdman (2016) examined the association between performance on three cognitive measures and FPD during downwind segments in circuit flight in 54 pilots. The two cognitive measures assessing multiple EFs and non-EF abilities were the Shifting Attention Task (i.e., number of perseverative errors) and a composite score based on several CogScreen-AE subtests. Both the Shifting Attention Task and the composite score based on CogScreen-AE subtests were significant unique predictors of FPD in a regression analysis (β = −0.216, *t* = −2.207, *p* = 0.032 and β = −0.337, *t* = −3.000, *p* = 0.004, respectively). Additionally, significant correlations were reported for the Shifting Attention Task (*r* = −0.324, *p* < 0.05) and the CogScreen-AE subtests composite score (*r* = −0.456, *p* < 0.01), demonstrating that a better performance on these measures was associated with a smaller FPD.

Causse et al. (2017) assessed the association between multiple EFs and non-EF abilities (measured with the One Touch Stockings task) and FPD during landing in 26 student pilots, while measuring functional near-infrared spectroscopy (fNIRS). They reported a non-significant correlation (*r* < 0.40).

##### Results: Flight path deviation and working memory updating

Causse et al. (2011a) used a multiple regression analysis and found that working memory updating performance on the Two-back task, together with logical reasoning ability and experience, significantly predicted FPD during take-off and reaching a specific waypoint [*F*_(1, 15)_ = 5.08, *p* = 0.04]. However, the correlation between working memory updating ability and FPD was non-significant (*r* = −0.35). In a next study, Causse et al. (2011b) found a significant medium experience-partialled correlation between working memory updating performance on the Two-back task and FPD during take-off and reaching a specific waypoint (*r* = −0.41, *p* = 0.022), explaining 17% of the variance. In contrast, in a more recent study, Causse et al. (2017) found a non-significant correlation between performance on the Spatial Working Memory test and FPD during landing (*r* < 0.40).

##### Results: Flight path deviation and conflict monitoring

Causse et al. (2011a) found that in a multiple regression analysis, conflict monitoring performance on the Spatial Stroop task could not predict FPD during take-off and reaching a specific waypoint. Additionally, the correlation between conflict monitoring performance and FPD was non-significant (*r* = 0.15). Similarly, Causse et al. (2011b) found a non-significant experience-partialled correlation between conflict monitoring on the Spatial Stroop task and FPD during take-off and reaching a specific waypoint (*r* = 0.19, *p* = 0.322, *R*^2^ = 0.03).

##### Discussion flight path deviation

Multiple EFs and non-EF abilities, as measured by the number of perseverative errors, was not a predictor of FPD and was neither associated with FPD (Causse et al., 2011a,b). In contrast, in the study by Van Benthem and Herdman (2016) the number of perseverative errors was both a predictor of FPD and significantly associated with FPD. The mixed results from these three studies could be due to differences in how FPD was assessed (i.e., angular deviations in the horizontal axis during take-off and reaching a waypoint vs. deviations from the specified altitude and airspeed during downwind segments of circuit flights) and/or due to differences in mean hours flown by the pilots (1,676 and 1,545 h vs. 557). Experience or expertise is a factor known to improve flying performance and to protect against aging effects in cognition (Taylor et al., 2007). Further findings illustrate the contributions of multiple EFs and non-EF abilities to FPD, though it is difficult to judge if and which specific EFs contributed to this association. Logical reasoning ability was a significant predictor of FPD during take-off and reaching a specific waypoint (Causse et al., 2011a), and showed a medium to large associations with FPD (Causse et al., 2011a,b). Contrary to this, another complex measure (measuring among others spatial planning and working memory) was not associated with FPD during a difficult landing condition in student pilots (Causse et al., 2017). This was probably because this test (i.e., One Touch Stockings task) is sensitive to brain dysfunctions and shows a ceiling effect in healthy young adults (Krikorian et al., 1994). Finally, a composite score measuring multiple EFs and non-EF abilities was a significant predictor of FPD during downwind segments of circuit flights, and was associated with FPD (Van Benthem and Herdman, 2016).

Studies taking specific EFs into account focused on working memory updating and conflict monitoring. First, working memory updating performance could predict FPD during take-off and reaching a specific waypoint in private licensed pilots (Causse et al., 2011a). Pilots with better working memory updating skills showed less deviations from the intended flight path. This could be expected as working memory updating supports the estimation of flying-relevant aspects (Chialastri, 2012). The association between working memory updating and FPD was only significant when controlling for flight experience (Causse et al., 2011a,b). However, no association was found between spatial working memory updating performance and FPD during a difficult landing condition in a sample of student pilots (Causse et al., 2017). Interestingly, in this study there were no associations between any of the employed cognitive measures and FPD. The mixed results could be due to discrepancies in the experience of the pilots and the modality and demands of the working memory tasks. Second, conflict monitoring performance was neither a predictor of, nor associated with, FPD during take-off and reaching a specific waypoint (Causse et al., 2011a,b). FPD seems to be an aspect of flying that involves little conflicting information, so a relationship between conflict monitoring and FPD was not necessarily expected. Conflict monitoring is more likely to be involved in situations where there is a sudden need to perform multiple tasks (i.e., troubleshooting). In these circumstances, several motor plans are simultaneously activated and pilots have to prioritize and sequence the actions needed. Other scenarios requiring conflict monitoring might entail reacting to unanticipated changes in the environment (e.g., inconsistent parameters, unexpected bad weather). However, there are only two studies on this specific EF, so further research is needed to draw firm conclusions about the involvement of conflict monitoring in FPD.

###### Comments for future studies

Again, important for future studies is to assess which specific EFs contribute to FPD. The tasks that were used in the included studies to assess perseverative errors are usually considered to be measures of cognitive flexibility (or as we call it set-shifting). However, these tasks involve multiple EFs, as well as non-EFs abilities. Additionally, tasks such as the WCST have the risk of floor effects in healthy participants with respect to perseverative errors, as they were originally developed to test for frontal lobe dysfunction. A key question in the assessment of actual set-shifting is which measures are most sensitive. For healthy participants, so-called switching costs (more precisely, the difference between trials with a repetition of a task compared to trials with a change of task in switching tasks) and preparation effects are popular measures of set-shifting in experimental psychology and cognitive neuroscience. Two fundamental observations in switching tasks are longer RT in switching conditions compared to repetition conditions (i.e., switching costs), and the ability to reduce switching costs with preparation by using cues (see for instance Karayanidis et al., 2010). These measures are well-suited for research in flying, as pilots are known to have good cognitive skills in comparison to the general population and application of these paradigms could contribute to the current knowledge base about switching costs and proactive abilities in pilots. For assessing specific EFs, such as working memory updating, conflict monitoring, response inhibition, and set-shifting, it is methodologically ideal to use multiple tasks to mitigate task-specific context dependency (as also noted in the methods section).

##### Results: Crosswind landing decision and multiple EFs and non-EF abilities

In the study by Causse et al. (2011a), 41.6% of the pilots erroneously continued landing despite unfavorable wind conditions. The number of perseverative errors on the computerized WCST did not significantly predict the correct crosswind landing decision in a discriminant analysis [β = −0.379, *F*_(1, 14)_ = 2.584, *p* = 0.142]. In contrast, Causse et al. (2011b) found that pilots who made the correct crosswind landing decision had significantly less errors on the computerized WCST, as compared to pilots making the incorrect decision [*F*_(1, 30)_ = 5.33, *p* = *0.0*27, ηp^2^ = 0.15]. Here, 50% of the pilots incorrectly persisted with landing. One could notice that the authors ran a one-way ANOVA with decision as a categorical variable whereas a Chi-square test would have been more relevant. However, we do believe that the reported results provide interesting insights to better understand the executive mechanisms underlying decision making. Neither study found that logical reasoning abilities as measured by the LDRT were related to a correct crosswind landing decision. Causse et al. (2011a) reported that logical reasoning abilities did not significantly predict correct crosswind landing decisions in a discriminant analysis [β = −0.144, *F*_(1, 14)_ = 0.486, *p* = 0.503], and Causse et al. (2011b) found no significant difference in logical reasoning abilities between pilots who made the correct vs. those making the incorrect crosswind landing decision.

##### Results: Crosswind landing decision and working memory updating

The study by Causse et al. (2011a) showed that working memory updating performance on the Two-back task could predict a correct crosswind landing decision in a discriminant analysis [β = 1.551, *F*_(1, 14)_ = 20.676, *p* = 0.001]. A model including working memory updating, experience, and motor impulsivity correctly classified 100% of the pilots who erroneously persevered with landing, and 91.6% of the pilots who correctly decided to go around. In the study by Causse et al. (2011b), pilots who made a correct landing decision scored significantly higher on the Two-back task as compared to those who made an incorrect decision [*F*_(1, 30)_ = 9.76*, p* = 0.003, ηp^2^ = 0.25] (see previous paragraph for our comment regarding the statistical analysis).

##### Results: Crosswind landing decision and conflict monitoring

Causse et al. (2011a) found that conflict monitoring performance on the Spatial Stroop task performance did not predict a correct crosswind landing decision [β = 0.264, *F*_(1, 14)_ = 1.072*, p* = 0.327]. Likewise, Causse et al. (2011b) did not find a significant difference in conflict monitoring performance on this task between pilots who made the correct and pilots who made the incorrect crosswind landing decision.

###### Discussion crosswind landing decision

Crosswind landing decisions are probably the most specific flying aspect and are considered as one of the most critical aspects of flying. Evidence shows that pilots have the tendency to continue landing despite adverse conditions and that poor crosswind landing decisions are the reason for most incidents (Ebbatson and Jarvis, 2004). The results show that working memory updating performance could significantly predict a correct crosswind landing decision (Causse et al., 2011a) and could dissociate pilots making a correct decision from those making an incorrect one (Causse et al., 2011b). Measures of multiple EFs and non-EF abilities and conflict monitoring did not appear to be predictors of a correct crosswind landing decision (Causse et al., 2011a), although pilots making a correct decision did show significantly less perseverative errors (Causse et al., 2011b). The finding that working memory updating ability is important in crosswind landing situations seems to be in line with the fact that pilots have to quickly and accurately calculate the crosswind component, incorporating aspects such as the current weather conditions and the aircraft specifications, in order to make a correct decision.

###### Comments for future studies

There were no studies looking into the relationship between crosswind landing decisions and set-shifting or response inhibition, making it an interesting direction for future research. Beyond standard switching tasks, examining switching abilities directly within a flying context can improve ecological validity. For example, the Multi-Attribute Task Battery (MATB-II) and even flying simulators can be adapted to include specific switching trials. In these trials, participants would shift between distinct tasks or adjust priorities based on new demands, such as moving from instrument monitoring to responding to an alert. This setup helps isolate task-switching moments in a flight-related environment, allowing for a detailed analysis of the cognitive demands associated with switching in aviation contexts.

##### Results: Flight ability and multiple EFs and non-EF abilities, inhibition and working memory updating

Zheng et al. (2022) investigated the relationship between multiple EFs and non-EFs and flight ability. They found that worse inhibition control (measured by the Hayling Sentence Completion Task) was significantly correlated with flight ability (*r* = −0.40, *p* < 0.05). Working memory updating (measured by the Corsi Block test) and multiple EFs and non-EFs (measured by errors on the WCST), did not significantly correlate with flight ability (*r* = −0.29, reported as *p* > 0.05; *r* = −0.14, reported as *p* > 0.05, respectively).

###### Discussion flight ability

Based on these results, inhibition appears to play a role in flight ability performance. However, this is the only study that assessed the relationship between inhibition and flying performance. Additionally, they only utilized correlational analysis, which does not account for any confounding variables. Moreover, the assessment criteria for flight ability were not clearly defined in the article, and the study sample consisted of pilots without any flying experience. These limitations make it difficult to draw definitive conclusions.

###### Comments for future studies

Exploring the role of response inhibition across different flight tasks and conditions, using a more robust statistical design (e.g., multiple regression analysis), can help clarify its importance. It is essential to conduct studies with clearly defined flight ability criteria and include pilots with varying levels of experience to provide a more comprehensive understanding.

#### Navigating

##### Results: Diversion management and multiple EFs and non-EF abilities

Van Benthem and Herdman (2017) assessed whether multiple EFs and non-EF abilities (measured with the Shifting Attention Task) could predict the performance on a diversion management task in a sample of 34 pilots. They found that diversion management was best predicted by the measure of multiple EFs and non-EF abilities, together with license type and prospective memory, in a multiple regression model, explaining 42% of the variance. They reported that the multiple EFs and non-EF abilities measure was the best predictor, but did not provide additional statistics in their paper. The multiple EFs and non-EF abilities measure also showed a significant correlation with the diversion management score (*r* = −0.527, *p* < 0.01). Van Benthem and Herdman (2021), however, did not find a significant correlation between multiple EFs and non-EFs (measured by the Shifting Attention Task) and performance on a diversion response task (*r* = −0.213, *p* > 0.05). Performance on the Shifting Attention Task, together with recent pilot-in-command hours (Recency Hours, 12 months) and age, explained 18% of the variance in diversion response scores, with the Shifting Attention Task score being the second most important predictor.

##### Results: Staying on course and multiple EFs and non-EF abilities

Taylor et al. (2000) examined the correlation between two composite scores measuring multiple EFs and non-EF abilities and the flight summary score component Staying on course. Both the composite score based on CogScreen-AE subtests and the Shifting Attention Task composite score were significantly correlated with the component Staying on course (*r* = 0.49, *p* < 0.05 and *r* = 0.23, *p* < 0.05, respectively).

##### Results: Alternate aerodrome errors and multiple EFs and non-EF abilities

Van Benthem and Herdman (2021) found that multiple EFs and non-EFs, measured by the Shifting Attention Task, significantly correlated with alternate aerodrome errors (*r* = −0.271, p < 0.05).

###### Discussion diversion management, staying on course and alternate aerodrome errors

Multiple EFs and non-EF abilities contributed to the prediction of a diversion management/response score (Van Benthem and Herdman, 2017, 2021). However, while Van Benthem and Herdman (2017) also found a significant association, this was not observed in the later study by Van Benthem and Herdman (2021). Van Benthem and Herdman (2021) did find a significant association between multiple EFs and non-EFs and alternate aerodrome errors. Additionally, significant associations were demonstrated between two different composite scores measuring multiple EFs and non-EF abilities and the flight summary score component Staying on course (Taylor et al., 2000). These results hint at a possible involvement of multiple EFs and non-EF abilities in navigation.

###### Comments for future studies

To determine whether and which EFs contribute to navigation, future research should focus on tasks measuring specific EFs. Several specific EFs seem relevant for adequate navigation of an aircraft, as it requires up-to-date situational awareness. Impaired situational awareness can lead to an episode in which pilots lose their ability to correctly evaluate the plane's position (Marquardt, 2019) and spatial disorientation remains a known factor that frequently contributes to flying incidents (Newman and Rupert, 2020). Especially working memory updating might be important for situational awareness, as it allows the pilot to adequately monitor the current position of the aircraft and predict possible changes.

#### Communicating

##### Results: Aviation communication and multiple EFs and non-EF abilities

Taylor et al. (2000) assessed the correlation between two composite scores measuring multiple EFs and non-EF abilities and the flight summary score component Dialling in communication frequencies. The composite score based on a combination of CogScreen-AE subtests significantly correlated with this component (*r* = 0.39, *p* < 0.05), as did the Shifting Attention Task composite score (*r* = 0.43, *p* < 0.05). Taylor et al. (2005) looked into the correlations between three cognitive composite scores and performance on an aviation communication task in a sample of 97 licensed civilian pilots with different levels of experience. The composite score based on the Shifting Attention Task is considered to be a measure of multiple EFs and non-EF abilities, and showed a significant correlation with performance on the aviation communication task (*r* = 0.43, *p* < 0.05). In the study by Kennedy et al. (2013), four cognitive composite scores, together with level of expertise, were assessed to determine whether they could predict Communication, a component of a flight summary score. In a mixed effects model, the two composite scores measuring multiple EFs and non-EF abilities turned out to be significant predictors of the Communication component: Shifting Attention Task composite score (β = 0.095, *p* = 0.021) and Pathfinder composite score (β = −0.266, *p* < 0.0001). As mentioned earlier the composite score based on a combination of CogScreen-AE subtests was not included in the model.

##### Results: Aviation communication and working memory updating

In the study by Taylor et al. (2005), a significant correlation was found between a *Working memory span* composite score and performance on an aviation communication task (*r* = 0.76*, p* < 0.05). Morrow et al. (2003) investigated whether verbal working memory updating (measured by the Sentence Span test) could predict aviation communication performance outside of a flight simulator in a sample of 91 pilots. For aviation communication the outcomes were instruction readback, route probe, and route recall. All three aviation communication outcomes were significantly predicted by verbal working memory updating, together with spatial ability, in a hierarchical regression analysis (*p* < 0.001): ATC message readback (β = 0.31), route probe (β = 0.34), and route recall (β = 0.38). Additionally, verbal working memory updating showed a significant correlation with all three aviation communication outcomes (instruction readback: *r* = 0.42, *p* < 0.001, route probe: *r* = 0.40, *p* < 0.001, and route recall *r* = 0.46, *p* < 0.001).

###### Discussion aviation communication

One study found that two composite scores measuring multiple EFs and non-EF abilities could predict aviation communication performance (Kennedy et al., 2013). Additionally, two studies assessed multiple EFs and non-EF abilities based on different tasks and composite scores and found associations with aviation communication tasks (Taylor et al., 2000, 2005). Interestingly, two studies that focused specifically on working memory updating demonstrated medium to high correlations with aviation communication tasks both in- and outside of a flight simulator (Taylor et al., 2005; Morrow et al., 2003). Verbal working memory updating was furthermore one of the factors predicting performance on a paper-and-pencil communication task (Morrow et al., 2003). Such findings were expected, as aviation communication strongly involves working memory (Durantin et al., 2016): pilots have to memorize information related to their speed, heading, and altitude from the air traffic control message, read back the details, and subsequently adapt their trajectory and speed. Air traffic control messages represent a common source of errors, as they tend to be misunderstood or even executed incorrectly (e.g., Molesworth and Estival, 2015). These hints for a relationship between working memory updating and aviation communication display an important finding, as direct training of working memory capacity and updating could have potential to prevent errors in aviation communication.

###### Comments for future studies

Future studies may investigate the most efficient way of delivering air traffic control messages with regard to working memory updating. Working memory updating tasks should focus on auditory or language -related modalities (for instance an auditory n-back task to better understand its role in communication aspects of flying. Other specific EFs, such as set-shifting, should also be assessed in future research, as this specific EF is expected to take place when the pilot has to shift from one task (e.g., navigating or programming the avionics) to the interaction with air traffic control, and subsequently execute the given instructions.

## General discussion

The objective of this study was to investigate the role of EFs in enhancing pilots' performance in flying, navigation, and communication. We conducted a review of twelve relevant studies that explored the contributions of both EFs and non-EFs in understanding various pilot abilities. Several studies reported mixed or contradictory findings, particularly regarding the relationship between EFs and basic flying skills among experienced pilots. This inconsistency is not surprising, as pilots often rely on automatic processes for routine tasks—such as maintaining heading, speed, altitude, and executing checklists. Consequently, traditional EFs frameworks demonstrate limited predictive power because EFs, such as working memory, may not be actively engaged during these routine operations.

However, EFs are critical in non-routine, unexpected, or complex scenarios that demand quick and adaptive thinking. As modern aviation increasingly relies on automation to handle routine tasks and maintain stable flight conditions—particularly as we move toward extended minimum crew operations—autopilot systems cannot easily replicate the controlled cognitive processes of human pilots. While autopilots are designed for predictable scenarios and function through automated processes similar to perception and action schemes, EFs become essential when pilots face unexpected challenges and must regain control in situations where autopilot systems fail. This is underscored by recent analyses of transportation accidents and experiments highlighting instances in which pilots failed to demonstrate adaptive behavior (Sarter and Woods, 1997; Dehais et al., 2015). Thus, EFs are vital for ensuring safety and performance in aviation. Although none of the studies we reviewed specifically examined pilot-automation interactions, some explored the EF framework in the context of decision-making and navigation, which require effective planning and adaptability, especially during in-flight emergencies or diversions. Our analysis of crosswind landing decisions and diversion management reveals clearer associations. For instance, research by Causse et al. (2011b) found that working memory skills significantly predict go-around decisions during crosswind landings. Similarly, Causse et al. (2011a) demonstrated that working memory and perseverative errors on the Wisconsin Card Sorting Test (WCST) predict diversion decisions. The work of Kennedy et al. also provides valuable insights into the influence of both EFs and non-EFs factors on successful diversion management.

Nonetheless, limitations in statistical reporting within these studies can impede comprehensive interpretations, thus affecting the validation of our EF framework. These findings align with a recent review by Dehais et al. (2019a), which investigated the neural correlates of perseverative behavior in pilots and reported evidence of deactivation in brain regions supporting executive functioning (e.g., the dorsolateral prefrontal cortex) when pilots displayed a lack of mental flexibility.

Finally, the studies we reviewed regarding the relationship between EFs and communication management between pilots and air traffic control (ATC) consistently demonstrate the predictive power of various working memory tasks on pilots' communication abilities. This supports the hypothesis that communication with ATC is a structured and procedural task requiring pilots to listen attentively and convey crucial information, such as wind direction, QNH (air pressure), visibility, and specific headings. The strong correlation between laboratory tasks and operational communication emphasizes the predictable relationships observed, especially when compared to more complex flying tasks that involve multiple interrelated EFs and non-EFs.

While it is important to conduct more studies to validate the overall framework of EFs in supporting pilots' performance, we discuss four critical issues that have general significance: limited emphasis on certain EFs, sample selection, statistical design, and the assessment of specific EFs.

Historically, research on EFs in aviation has primarily focused on functions like working memory that is more easily linked to critical aspects of flight performance, such as maintaining situational awareness and managing complex information flows. In contrast, EFs like set-shifting, conflict monitoring and response inhibition are often understudied, despite their theoretical importance. This discrepancy likely stems from the inherent complexity in designing tasks that isolate these functions within the dynamic and multifaceted context of flight scenarios. Measuring set-shifting and response inhibition in high-fidelity simulations or real-time flight conditions is particularly challenging because these functions frequently interact with other cognitive demands in complex ways. As a result, it becomes difficult to separate their specific contributions to flight performance from the influence of overlapping cognitive processes, which limits our understanding of their distinct roles in aviation settings.

To address the research gaps surrounding the EFs of set-shifting and response inhibition in aviation, various methodologies and technologies can be effectively utilized. Customized flight simulators with scenarios that demand rapid task-switching or sudden inhibition of actions—such as last-minute runway changes or go-around maneuvers—could specifically target these EFs. While fMRI studies have shown some potential for examining neural correlates of flying performance, their application is limited by the need for simplified tasks (Durantin et al., 2017; Adamson et al., 2014; Causse et al., 2013; Dehais et al., 2020a). In contrast, neuroimaging tools like functional near-infrared spectroscopy (fNIRS) and electroencephalography (EEG) allow real-time observation of brain activity, particularly in the prefrontal cortex, within more realistic settings such as simulator (Gateau et al., 2015) and real flight conditions (Gateau et al., 2018). Furthermore, embedding cognitive tasks such as the Stop-Signal Task or Task-Switching Paradigm in simulations provides controlled yet dynamic measures of response accuracy and speed. Complementary technologies, including eye tracking and heart rate variability monitoring, can further enrich data on attention distribution and stress responses. These integrated approaches offer valuable insights into these understudied EFs, with the potential to enhance pilot training and assessment.

The inclusion of pilots with different licenses and different flight experiences is essential for a fundamental study of the relationship between EFs and flying performance. For example in the study by Taylor et al. (2000) the composition of the sample of civilian pilots included: 23% private-licensed pilots rated for visual flight conditions, 66% non-air-transport instrumented-rated pilots, and 11% held air-transport ratings. Such an approach has the advantage that both pilots selected for their cognitive abilities (e.g., professional pilots) and those not selected for their cognitive abilities (e.g., hobby pilots) can be evaluated. With such an approach, the relationship between EFs and flying performance can be better assessed because the sample has a larger variance in cognitive performance than a highly preselected group where a relationship might disappear. However, the majority of the studies did not include different types of pilots in their sample, limiting the generalizability of the results.

Another important point to adequately investigate the relationship between EFs and flying performance is the statistical design. Many of the results extracted from the available studies refer to correlations, but caution is needed when interpreting them. Simple correlations between independent and dependent variables can be misleading if confounding variables play a role. Thus, multiple regression analyses are often more meaningful because several predictors are taken into account. However, when predicting flying performance based on different (cognitive) predictors using multiple regression analysis, intercorrelations between predictors must be taken into account when interpreting the results. This is because the β-coefficients may be influenced by both the correlation of the predictors with the dependent variable and the intercorrelation between the predictors (i.e., multicollinearity). An example would be the prediction of flying based on intelligence, EFs, and attention; because all these variables are intercorrelated (Van Aken et al., 2016). To determine the significance of the individual predictors in a multiple regression analysis, the partial or semipartial correlations are needed. These values provide information about the relative importance of the predictors by showing how much they uniquely contribute to the *R*^2^ not accounted for by the other predictors. Unfortunately, the majority of the studies did not report this information.

Finally, for the assessment of specific EFs, such as working memory updating, conflict monitoring, response inhibition, and set-shifting, it is methodologically ideal to use multiple tasks. For example, the Flanker, Simon, and Stroop tasks could be used to assess conflict monitoring. Using multiple tasks for a given EF can alleviate the problem of task-impurity. This problem arises from the fact that tasks are embedded in a context and functions can never be measured purely. Performance on any task always encompass several factors, for instance a specific EF factor (e.g., conflict monitoring), a common EFs factor, non-EFs processes (e.g., visual processing), and an error component (e.g., tiredness) (see Snyder et al., 2015).

## Directions and future research

Cognitive functioning has long been recognized as a vital component in pilot selection, training, and maintaining safe flying performance, with EFs playing a critical role. This review shows first support that various EFs and non-EF abilities are involved in several aspects of flying and are especially crucial for specific challenges, such as the use of working memory (updating) in making critical decisions like landing and diversion, as well as in aviation communication.

However, the review clearly demonstrates that research in terms of the different flying aspects and specific EFs is still in its early stages as few studies concretely assessed this relationship. For instance, set-shifting and response inhibition is not assessed with specific tasks such as switching and stop-signal tasks respectively, although the relation is theoretically considered important. So far, we have highlighted the methodological challenges for EF tasks (multiple EFs, specific EFs, non-EFs abilities) and interpreting the performance on such tasks. Additionally, it is important to consider characteristics like personality traits (Breuer et al., 2023; Behrend et al., 2017), which can significantly affect performance in high-emotion contexts, such as crew management or situations involving uncertainty. These influences may not be fully captured by traditional EF assessments.

Therefore, further studies are needed to develop a more comprehensive framework that formalizes the cognitive task analysis of flight tasks. This framework should aim to develop a theory-based methodology for determining the roles of EFs and cognitive processes in specific flying situations, akin to the approaches seen in Naturalistic Decision Making research (Klein et al., 1986). Table 4 gives an example for a first basis of tasks and the cognitive task analysis during the visual approach to landing of a real flight of a B757 aircraft [adapted from Keller et al. (2003)]. This flight phase is considered to be particularly complex and high in workload and includes various cognitive processes, their interaction, and control by EFs.

**Table 4:** Tasks and Cognitive Task Analysis and possible involved cognitive processes in Visual Landing Approach of a B757 aircraft [adapted after Keller et al. (2003)].

Flight phase description	**The approach to landing phase** starts at the lower end of the descent and ends with the wheel touchdown in the landing phase. The aim of the approach is to bring the aircraft from intermediate altitudes, as prescribed, to a position, speed and configuration from which the pilot can land his aircraft. Pilots follow published approach procedures that determine the course, altitudes, and speed to a specific runway.
	**A visual approach** can be performed when pilots can see the airport and the runway from a distance. Further approval is given for the following steps. Pilots often follow other aircraft in the landing sequence, but must keep their aircraft far enough away, avoid terrain and obstacles, and point their aircraft toward the runway. In order to align the aircraft with the runway, special lighting systems ensure lateral and vertical guidance. The pilot decides on courses and heights to face.
	**The pilot's displays & controls**, in conjunction with the radio information and the view out of the window, provide all the information required for a safe approach. They are used during the approach by the pilots. Examples include the mode panel for selecting control and steering modes to change the flight path as needed; the primary flight display as a tool for attitude; and the navigation display, which provides pilots with a map view of the area.
Tasks	**Sequential tasks** include for a general approach: (a) Following air traffic communication, (b) Setting radio frequencies, (c) Deciding whether to engage automated flight control, (d) Maintain airspeed by monitoring airspeed indicators, (e) Adjusting the flaps by moving the flap lever to the correct position with one hand and observing the flap indicator on the front instrument panel, (f) Monitoring the localizer and glide slope, (g) Lowering the landing gear, (f) Using the arm speed brakes with a lever to deploy speed braked or spoilers, (h) Setting missed approach altitude to climb in the event of a missed approach, (i) Monitoring the altitude below 2,500 Feed AGL, (j) Executing the Before Landing Checklist to verify critical tasks that have to be completed prior to landing (k) Switching on landing lights, (l) Monitoring the descent rate, (m) Disengaging autopilot, (n) Flying manually using both hands, feet, while visually scanning the instruments and looking out the front window, as well as putting attention to the radio, (n) Approaching the aircraft by pulling the yoke while flying over the runway to bring the aircraft into the landing attitude
	**Non-sequential tasks** Monitoring the flight path and progress which involves scanning of the instruments, Double-checking and verifying of the altitude, speed and flaps throughout the approach, Monitoring the radio involves listening for communications, Monitoring of the aircraft systems
Cognitive task analysis	**Decision making** Several important decisions must be made during this phase of flight, e.g., should a missed approach be made because: there is an instruction from air traffic control, there is a glide slope that is too high or too low, flying too far to the left or right of the extended runway centerline, the speed is too high, the runway cannot be approached due to poor visibility and other weather-related events.
	**Approach problems and error shooting** might arise during this approach, e.g., aircraft-to-aircraft spacing errors because air traffic control prescribes a certain speed when the crew should actually slow down, furthermore distractions, e.g., when a high communication volume on the radio distracts the crew from other tasks because they are trying to understand all relevant information.
	**Situational awareness:** Pilots must continuously have a mental picture of where the aircraft is at any given moment, whether the flight is going according to plan and how any changes made will affect the rest of the flight plan.
*Involved cognitive processes*	**Possible examples are:** • Motor coordination • Performance monitoring (conflict monitoring, error monitoring) and comparison with given and internal goals • Decision-making • Control and use of attention • Shifting attention between modalities (visual, auditory) • Multitasking, deciding which task engage and when, switching between tasks • Spatial working memory (updating) • Prediction

Based on the findings we propose the following general directions for future research:

• *Conceptualize measures of EFs and flying performance based on existing theorie*s. We suggest the framework EF-Fly which is based on the model of Miyake et al. (2000) for EFs and the hierarchical rule of flying performance (“fly, navigate, and communicate”).

• *Use sensitive tasks to assess specific EFs*. We advise to include tasks that measure specific EFs (i.e., working memory updating, set-shifting, response inhibition, and conflict monitoring), as opposed to complex tasks that measure multiple EFs as well as non-EF abilities. The administration of multiple tasks per specific EF is also recommended. Finally, it is important to include tasks of an appropriate difficulty level for the research population. It is discouraged to use EFs tasks that were developed for the assessment of severe impairments in clinical populations, as these would be too easy for high-functioning pilots and result in ceiling-effects.

- *Employ portable and simultaneous (neuro)physiological measures*: It is now possible to use portable brain imaging techniques, such as fNIRS or EEG systems, to investigate cognition in a simulated and a real cockpit in real-time (e.g., Dehais et al., 2019b). Wireless data collection enables monitoring brain activity in more naturalistic environments and makes it easier to capture more ecologically valid data. This neuro-ergonomic approach could help to better understand the neural mechanisms underpinning EFs (e.g., frontal midline theta oscillations and N2/P3 complex) in dynamic real-life situations.

• *Identify and validate cognitive processes in flight tasks* (real, simulated aviation) including the characterization of cognitive functions as a basis for a theory-based approach for identifying the cognitive functions in different flight tasks, and consequently design EFs-tasks in a flying context.

• *Assess flying performance and EFs in simulated or real flights or using immersive virtual reality*. The use of flight simulators is currently the standard in most flying research, enhancing the ecological validity. Predefine trials in which specific EFs are required and log when EFs tasks are required for later assessment of context-specific EFs. Further progress could be made in the standardization of flying performance measures. For example, FPD is currently measured in different scenarios (e.g., during take-off, landing, downwind) and calculated differently across studies (e.g., only vertical deviation or both vertical and horizontal). Using the same approach to assess FPD would ease the comparison of results. Additionally, a representative microworld task could be used to assess flying performance, since flying strongly involves system monitoring and management and is affected by many factors that are difficult to control (for example the Multi-Attribute Task Battery (Santiago-Espada et al., 2011).

• *Control for or take into account moderating/mediating factors* that could play a role in EFs, flying, or both. Factors such as age, experience, gender, and personality are shown to have an effect on executive functioning and/or flying performance. For instance, age-related cognitive decline may compromise the pilot's executive functioning (Kennedy et al., 2013), possibly having different effects on males and females (Baker et al., 2001). On the other hand, experience can be regarded as a factor protecting against cognitive decline (Causse et al., 2011b). Evidence also illustrates the importance of specific personality traits, such as impulsivity. Impulsivity predicts risky decision-making and is not influenced by training or a highly procedural environment (Behrend et al., 2017).

• *Investigate solutions to improve executive functioning* and thus flight safety. For instance, neurofeedback has shown promising results in enhancing EFs (Enriquez-Geppert et al., 2013, 2014). Alternatively, transcranial direct current stimulation is a neuromodulation technique that can be used to boost executive functioning (see Dehais et al., 2020b). Such training approaches have the advantage to directly target the neural underpinnings of cognition and therefore enhance the probability for long-term effects and generalization, rather than to affect cognition indirectly via games and computerized training. To date, very few studies have investigated the use of such brain training techniques for aviation. There is a definite need to assess their efficiency to improve cognition in real-life scenarios. Further solutions to be assessed are aerobic fitness training, mindfulness meditation and sleep management. Design and assess comprehensive EFs enhancement program embedded within standard training schedules, in airlines and training institutes.

## Practical applications

Understanding the precise nature of the relationship between EFs and flying performance has the potential to fuel research on the practice of pilot selection and cockpit design, and to influence brain training approaches to enhance pilots' cognition in order to improve overall flight safety.
